# dFmr1 Plays Roles in Small RNA Pathways of *Drosophila melanogaster*

**DOI:** 10.3390/ijms18051066

**Published:** 2017-05-16

**Authors:** Valeria Specchia, Simona D’Attis, Antonietta Puricella, Maria Pia Bozzetti

**Affiliations:** Dipartimento di Scienze e Tecnologie Biologiche ed Ambientali (DiSTeBA)—University of Salento, 73100 Lecce, Italy; valeria.specchia@unisalento.it (V.S.); simonadattis@libero.it (S.D.); antonietta.puricella@gmail.com (A.P.)

**Keywords:** FMRP/dFmr1, fragile-X syndrome, piRNA pathway, *crystal-Stellate* system, dFmr1 interactors

## Abstract

Fragile-X syndrome is the most common form of inherited mental retardation accompanied by other phenotypes, including macroorchidism. The disorder originates with mutations in the *Fmr1* gene coding for the FMRP protein, which, with its paralogs *FXR1* and *FXR2*, constitute a well-conserved family of RNA-binding proteins. *Drosophila melanogaster* is a good model for the syndrome because it has a unique *fragile X-related* gene: *dFmr1*. Recently, in addition to its confirmed role in the miRNA pathway, a function for dFmr1 in the piRNA pathway, operating in *Drosophila* gonads, has been established. In this review we report a summary of the piRNA pathways occurring in gonads with a special emphasis on the relationship between the *piRNA* genes and the *crystal-Stellate* system; we also analyze the roles of dFmr1 in the *Drosophila* gonads, exploring their genetic and biochemical interactions to reveal some unexpected connections.

## 1. Introduction

### 1.1. The Fragile-X Mental Retardation Gene

*dFmr1* is the *Drosophila* homolog of the gene responsible for fragile-X mental retardation syndrome, one of the most frequent inherited causes of human mental retardation. Humans have three homologous genes coding for the Fragile-X Mental Retardation Proteins: *Fmr1*, *FXR1*, *FXR2*, with high sequence similarity; all of them code for RNA-binding proteins [1,2,3]. Mutations in *Fmr1*, located on the X chromosome and coding for FMRP proteins, are responsible for the syndrome. FMRP and its autosomal paralogs, the Fragile X-Related proteins FXR1P and FXR2P, constitute a conserved, small family of RNA-binding proteins. FMRP has been studied in humans, mice, and *Drosophila*, principally in relation to its role in the nervous system, at the synapses, as a translational regulator working with different hypothesized mechanisms: (i) by repressing translational initiation [4,5,6]; (ii) by the miRNA pathway binding to the 3′ end of target mRNAs [7,8,9,10,11,12]; and (iii) by direct interaction with translating ribosomes [8,12,13,14,15]. The human FMRP is expressed in different tissues during development, with a preference for the gonads and the brain and, as expected, the main effects of its loss are in the brain and the testes [16].

**Figure 1:**
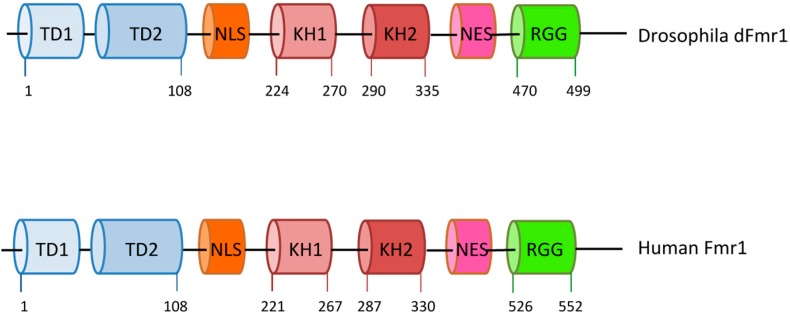
Domain organization of *Drosophila* and human Fmr1. The drawings are not to scale; the exact positions of the amino acids are indicated. TD1 and TD2 are tudor domains; NLS stands for nuclear localization signal; NES stands for nuclear export signal; KH1, KH2, and RGG motif are indicated.

*Drosophila melanogaster* is considered a good model for fragile-X syndrome [17] because it has a single, well-conserved *fragile X-related* gene named *dFmr1* [18,19], exhibiting high sequence similarity with all three human genes, and is predicted to be ancestral to the three mammalian members. *dFmr1* mutant flies exhibit defects in neuronal structure and function, and in germline development, resembling those observed in *Fmr1* mutations in mice and humans [19,20]. The human FMRP and *Drosophila* dFmr1 exhibit similar structures and domains [21]; they contain two KH domains, two Tudor domains, and an RGG box, in addition to a nuclear localization signal (NLS) and a nuclear export signal (NES) [2,3,21,22,23,24] (Figure 1). The high conservation between the two proteins suggests a common molecular role that is also supported by the phenotypes in the mutants.

FMRP has a wide spectrum of functions; however, its main role is exerted in different small RNA-mediated pathways in humans and in the mouse and *Drosophila* models. The majority of the studies regarding the small RNA pathways in *Drosophila* have been conducted in the ovaries, leading to the identification of specific molecular functions of dFmr1.

### 1.2. Drosophila Small RNA Pathways: An Overview

Different classes of small RNAs have been described and characterized in the last two decades; they have different biogenesis and functions and belong to different small RNA pathways depending on which Argonaute protein they interact with. Small RNAs bind to specific Argonaute proteins, forming different RISC complexes that initiate pathways leading to different post-transcriptional and transcriptional target regulation [25]. The three main small RNA pathways, also called RNA interference (RNAi) pathways, are: (i) the siRNA pathway (and the related endo-siRNA pathway) that uses a small RNA molecules of 21–23 nucleotides and Ago2 as its dedicated Argonaute protein [26]; (ii) the miRNA pathway that uses small RNAs as long as the siRNAs, and exhibit a very peculiar biogenesis; in *Drosophila* the miRNAs are generated, via a two-step process, from endogenously transcribed primary miRNAs. miRNAs guide Ago1, the Argonaute protein predominantly involved in this pathway, to obtain a translational repression of the mRNA targets [27,28]; (iii) the piRNA pathway that was first discovered in the gonads, and was predominantly considered the gonadal-specific pathway [29,30,31,32] even though, recently, the piRNA pathway has also been found in the nervous system not only in *Drosophila* but also in mice [33,34]. Piwi-interacting RNAs (piRNAs) are longer than the other classes of small RNAs (23–32 nt long) and Piwi, Aubergine (Aub), and Ago3, defined as the Piwi subfamily proteins, are the Argonaute proteins involved in this silencing pathway. The piRNAs protect animal cells from the de-regulation of transposons and other repetitive genetic elements, preserving genome stability [35,36]. Although the three main RNAi pathways exhibit specific features and components, there is some evidence that they share some components or functions with other pathways related to the RNA metabolism [37,38,39,40,41,42].

### 1.3. piRNA Genes Are Conserved during Evolution

Piwi proteins and the piRNA genes have been identified in model organisms and also in humans. They are deeply conserved in evolution even though the molecular mechanisms in which they are involved are not completely understood. The mouse is the animal in which many studies have been conducted, demonstrating that the piRNA-mediated silencing of transposons is active in the male germline to ensure fertility [43]. The role of piRNAs and the conservation of peculiar elements belonging to the piRNA pathway, such as the Piwi proteins, Vasa, Maelstrom, MOV10L1/Armitage, Tudor domain proteins, and others, suggest that they may act to protect animal cells from transposable elements, keeping them silenced and ensuring genome stability in the gonads [36,44,45,46,47,48]. A comprehensive analysis and annotation of human piRNAs in adult human testes has revealed a relationship between the small RNAs and transposons [49]. However, the direct role of the piRNA pathway in the silencing of TEs has not been clarified so far. A link between the Piwi human genes (PIWIL1, PIWIL2, and PIWIL4) and the transposons has been indirectly demonstrated in the tumorigenesis of human testes. In particular, the epigenetic inactivation of the Piwi proteins and the Tudor protein TDRD1 causes a reduction in piRNA expression in addition to the DNA hypomethylation of LINE1, an active human transposon [50].

## 2. The piRNA Pathway in *Drosophila* Gonads

The piRNA-mediated pathway is involved in the transcriptional and post-transcriptional silencing of the transposable and repetitive elements of the genome. It occurs predominantly in the germ cells and in their somatic precursors in the gonads of both sexes and is required for fertility not only in flies but also in mammals [35,51]. Most of what has been found on the piRNAs biogenesis, their roles, and the piRNA-related genes in *Drosophila* comes mainly from a combination of genetics and deep sequencing approaches. Genome-wide screens together with transcriptomic analyses have been performed with the aim to identify as many piRNA-related genes as possible [52,53,54]. Both germ cells and their somatic precursors use the piRNA pathway to silence transposable elements (TE). The biogenesis of piRNAs starts with the transcription of specific genomic clusters located at different positions in the genome; they are composed of repetitive, non-functional relicts of transposable elements [29]. Two main types of piRNA clusters have been described, both transcribed by RNA Polymerase II: uni-strand clusters, like AT-X1 and cluster 2 (germline specific) and flamenco (somatic specific), transcribed as single-strand precursors; and dual-strand clusters, like 42AB and 80EF (germline-specific) [55,56,57], transcribed from both strands [29,31,51]. Clusters produce RNA precursors that are processed into piRNAs in the perinuclear region of the gonadal cells called “nuage”, which surrounds the nuclear envelope [58,59,60,61,62]. Although the roles of the piRNA-related genes are almost the same in the gonads of both sexes, some differences exist [63,64].

### 2.1. The piRNA Pathway in the Ovary

Most of the knowledge about the piRNA pathway comes from studies in the *Drosophila* ovary. After their transcription, the piRNA precursors enter different pathways depending on which piRNA will be produced and in which type of cell it will function, somatic or germline. In the somatic primary piRNA pathway occurring in the follicle cells, the piRNA precursors undergo a cleavage by the endonuclease Zucchini, which seems to have a role in generating both the 5′ and the 3′ end of the primary piRNAs [29,40,65,66,67]. They possess a strong 1U bias and, after their production, they are bound to Piwi and to other proteins (Helicases and Tudor domain proteins) involved in the pathway and located in the structure called the Yb-body [68]. After that, mature piRNAs enter the nucleus to exert TE transcriptional silencing [65,66,69,70,71,72]. The interaction between the Tudor protein Yb, one of the key components of the Yb body, and the RNA helicase Armitage have a fundamental role in the entrance of Piwi into the nucleus [72], where it is involved in the H3K9me3-mediated transcriptional silencing of transposons. After their transcription, the primary piRNA intermediates place themselves in a well-defined structure called a flam-body or Dot Com adjacent to the Yb-body [73,74].

The primary pathway also occurs in the germ cells, nurse cells, and oocyte, producing primary piRNAs. The immature piRNAs transcripts are transported by the DEAD box helicase UAP56 [75,76], to the perinuclear region of the cytoplasm, the nuage, where a multi-protein perinuclear complex operates. The Piwi clade proteins Aubergine (Aub) and Argonaute-3 (Ago3) are then bound to the primary piRNAs and enter the germline specialized “ping-pong” amplification cycle with these two proteins as protagonists in the nuage to produce the secondary piRNAs that silence TEs [29,35,51,77,78]. The piRNAs produced by the ping-pong amplification loop show conserved specific signatures, like A in 10th position of the RNA (10A bias) [51]. Piwi seems not to be involved in the germline-specific primary pathway [51,79,80,81,82].

Many proteins involved in piRNAs biogenesis and transposon silencing, such as Aubergine, Ago3, Vasa, Krimper, Tudor, Spindle-E, Tejas, and Kumo/Qin are localized in the nuage [51,61,83,84,85,86,87,88]. Although for some of these proteins the molecular mechanism of the piRNA pathway is not completely clarified, the complex framework of interactions and actions continues to be enriched with new discoveries. The Tudor protein Krimper, for instance, has a key role in the formation of the nuage, leading to an interaction between Aub and Ago3 [87,89], even though its role seems to be different in the piRNA pathways of ovaries and testes, where it is required in association with Aubergine for the proper localization of itself and Aubergine at the nuage [63,64].

### 2.2. The piRNA Pathway in the Testes and Its Role in the crystal-Stellate Regulation

**Figure 2:**
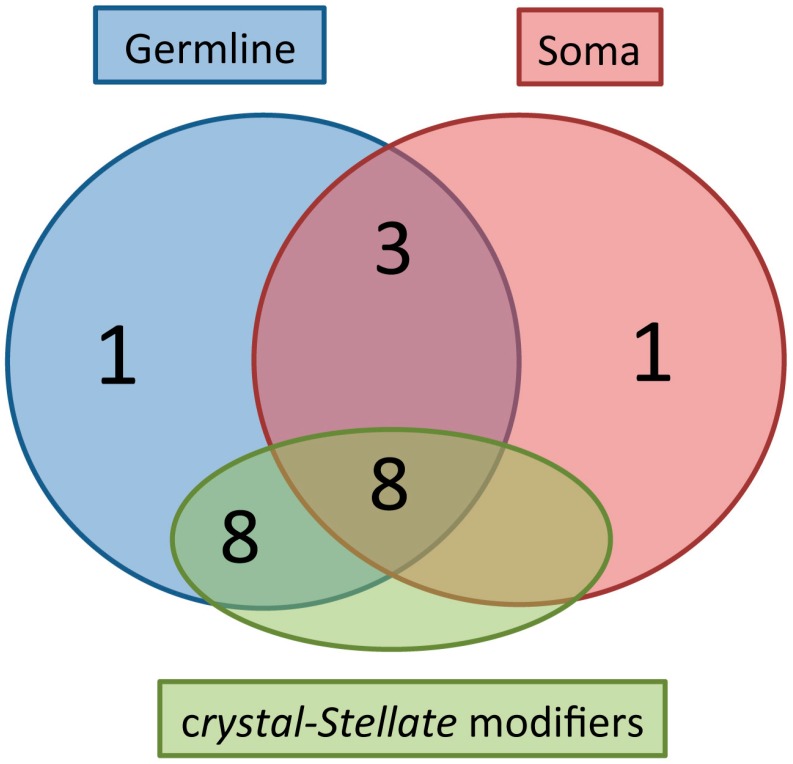
The majority of the germline and somatic piRNA genes are *crystal-Stellate* modifiers. A Venn-like diagram is reported, showing the distribution of the 26 piRNA genes listed in Table 1, in three groups: the “Germline” group represents the piRNA genes with a localization in the germline; the “Soma” group represents the piRNA genes with a localization in the somatic part of the gonad; the “*crystal-Stellate* modifiers” group represents the genes with a role in the silencing of the Stellate sequences. The distribution of the piRNA genes in the three groups is reported below: Germline (1) -> *cutoff*; Soma (1) -> *Yb*; Germline + Soma (3) -> *piwi*, *tudor*, *rhino*; Germline + *crystal-Stellate* modifiers (8) *-> aubergine*, *ago3*, *vasa*, *spindle-E*, *tapas*, *tejas*, *squash*, *capsuleen.* (*UAP56* has not been analyzed in relation to the *crystal-Stellate* regulation). Germline + Soma + *crystal-Stellate* modifiers (8) -> *ago1*, *armitage*, *krimper*, *zucchini*, *qin/kumo*, *dFmr1*, *shutdown*, *hsp83.* (*vreteno*, *papi*, *eggless*, *rhino*, *maelstrom*) have not been analyzed in relation to the *crystal-Stellate* regulation.

The “*crystal-Stellate* system” was first described as an example of heterochromatin–euchromatin interaction [90,91]. *Stellate* and *crystal* or *Su*(*Ste*) are homologous repetitive sequences located mainly at three different positions on the sexual chromosomes: *Ste*llate repeats are located on the X chromosome, at the euchromatic 12E region and at the heterochromatic *h26* region; *crystal* is on the Y at *h11* region. All of them are repetitive homologous sequences differing in some features [91,92,93,94,95]. Males lacking the *crystal* region exhibit crystalline aggregates in their spermatocytes [96,97]. In addition to crystals, these males also show defects in chromosome condensation and segregation. The *Stellate* and *crystal* loci are normally silent; however, deficiencies in the *crystal* region lead to the de-repression of the *Stellate* sequences in the germline. At the molecular level, the loss of the *crystal* region results in the production of a testes-specific *Stellate* mRNA of 750 bases in length [93,98,99]. In 1995 we discovered that the Stellate protein, produced by the *Stellate* mRNA, is the main component of crystalline aggregates [100]. An early genetic screening permitted us to identify some modifiers of the *crystal–Stellate* interaction among which are *aub^sting^* and *hsp83^scratch^* [98,99,101]. In 2001 it became clear that the *crystal-Stellate* regulation occurs by the small-RNA-mediated pathway [30,102,103], which, later, was identified as the specialized piRNA pathway [29,35,51,64,83,95]. In fly testes the most abundant piRNAs associated with Aubergine and Ago3 correspond to “*crystal*” piRNAs [83]. This finding has reinforced the relationship between *crystal-Stellate* regulation and the piRNA pathway. Indeed, the de-regulation of the *crystal–Stellate* interaction represents a simple readout to identify genes involved in the piRNA pathway in testes [95,98,99,101,104,105]. Many genes have been assigned to the piRNA pathway, and the majority of them came from studies in ovaries [51,77,106,107]. Even though no system-wide screening has been performed to date in searching for piRNA-mediated genes in testes, our group and others analyzed a wide variety of mutations of piRNA genes in relation to their role in the *crystal–Stellate* interaction. The main features of several piRNA genes identified during the years are summarized in Table 1, where their domains, functions, localization, cellular sub-localization, and especially their relationship with the *crystal-Stellate* regulation are highlighted [32,35,38,53,57,61,64,65,66,69,70,71,73,75,76,78,81,82,83,84,85,86,88,95,99,101,104,105,108,109,110,111,112,113,114,115,116,117,118,119,120,121,122,123,124,125,126,127,128]. From the analysis of the table and from Figure 2, where a Venn-like diagram is reported, showing the distribution of the genes listed in Table 1, it is clear that all the *crystal-Stellate* modifiers are piRNA-related genes, although the opposite is not true. A crucial point regards Piwi that is not involved in the piRNA-mediated silencing of *Stellate* in the testes: nonetheless, it is fundamental to piRNA biogenesis in the ovaries. Since Piwi seems not to be required in the germline primary pathway, at least in the ovary [64,79,80,81,82,129], it is conceivable that the silencing of the *Stellate* sequences mainly requires the germline primary pathway; this is also supported by the observation that the *Stellate*-related piRNAs share some signatures of the ping-pong pathway [64,83,95,128]. However, many genes required for the ping-pong amplification in the ovary are also required for the silencing of the *Stellate* sequences and of the transposons in testes (Table 1). Furthermore, the observations that Piwi and Rhino are required in the first step of the piRNA biogenesis in the ovaries [82] and that both proteins are not required for *Stellate*-related piRNA biogenesis in testes indicate that distinct pathways occur in the gonads of both sexes (the mechanism of primary piRNA production in the germline could be distinct from the piRNA production in the ovary) [63,87,89].

## 3. dFmr1 Participates in the piRNA Pathway Occurring in Gonads

Recently our group has demonstrated that dFmr1 plays a role in the piRNA pathway in testes and ovaries of *Drosophila melanogaster* [105]. This study originates from the observation that dFmr1, a protein involved in RNA metabolism, was found to be associated in a complex with components of the RNA interference pathway [7,8]. In addition, the complexity of the symptoms exhibited by fragile-X patients and by *Drosophila* mutants in the gonads and in the nervous system, along with the discovery of piRNAs in the nervous system of *Drosophila* and humans [33,34,130,131,132,133], allowed us to hypothesize that much more remains to be discovered about the multiple functions of the FMRP protein.

### 3.1. dFmr1’s Role in the Testes

**Figure 3:**
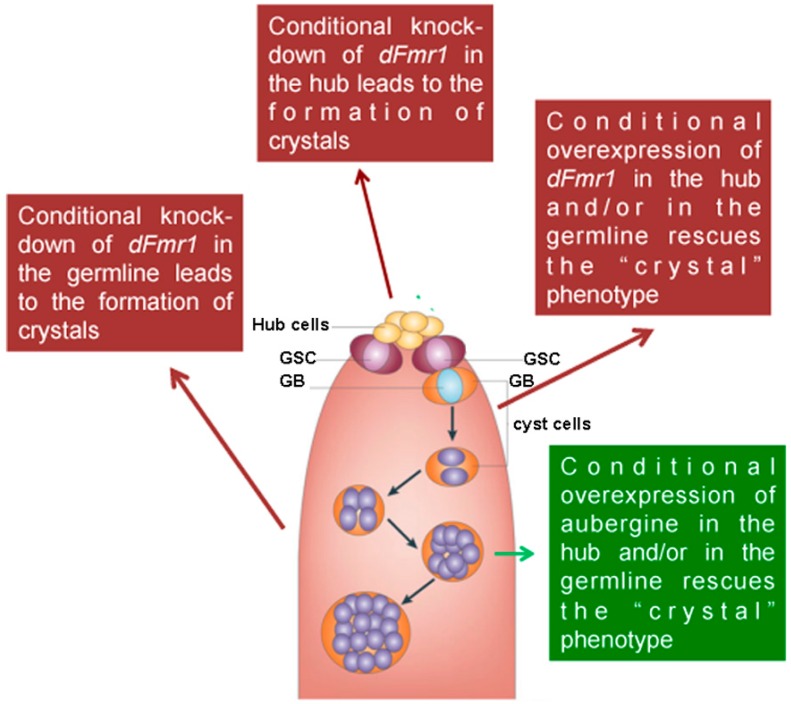
Germline and somatic requirement of dFMR1 in the testes. A scheme of the apical part of a testis is reported. Red arrows and boxes indicate the effect of the knockdown and/or the overexpression of dFmr1 in the hub and in the germline obtained using *upd*Gal4 (hub) and *nanos*Gal4 (germline) drivers. The green arrow and box indicates the effects of the overexpression of *aubergine*. GSC stands for germ stem cells; GB stands for gonialblasts.

**Figure 4:**
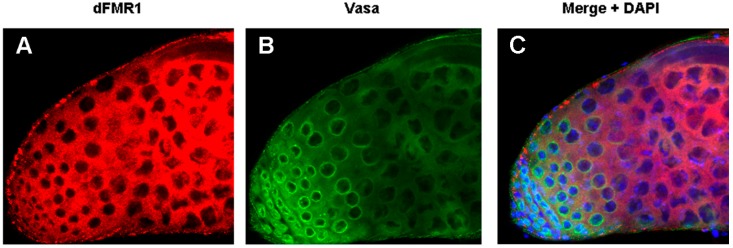
dFmr1 and Vasa immunolocalization in wt adult testes. Max intensity of triple confocal sections (40×) of a wild type testis labeled with (**A**) anti-dFMR1, (**B**) anti-Vasa, (**C**) Merge with the DAPI channel.

The role of dFmr1 in the piRNA pathway was demonstrated at first, by the presence of the *Stellate*-made crystalline aggregates in spermatocytes of *dFmr1***^∆^***^50^*, *dFmr1***^∆^***^113^* mutant males and in RNAi-*dFmr1* males in which the function of the protein was selectively reduced in different cells of the gonads (germinal or somatic) (Figure 3, red boxes). The presence of crystals was accompanied by the drastic reduction of the *Stellate*-related piRNAs. The confirmation of the role of dFmr1 in the piRNA pathway in testes, came from the observation that in *dFmr1* mutants and *dFmr1*-RNAi males, several transposable elements are activated with effects on the fertility of the individuals [105]. dFmr1 is expressed in the germ cells of adult testes as concluded from its colocalization with Vasa, a germline-specific marker [86,134], as previously described [105]. It is also present in the piNG bodies (Figure 4A–C), representing specific giant bodies of the nuage, where some piRNA components perform their function [135]. dFmr1 localization is predominantly cytoplasmic, even though a nuclear localization and function have been reported [136,137].

dFmr1 was also found to interact genetically and biochemically with two Argonaute proteins: Aubergine (a Piwi protein) (Figure 3, green box) and Ago1 (an Ago protein), both of which are located at the nuage of the germ cells. The genetic interaction with Aubergine and Ago1 also occurs in the nervous system at the neuromuscolar junctions [105].

The presence of the Tudor/Agenet domain in dFmr1 suggests that it may bind the Argonaute proteins by their symmetrically di-methylated arginines (sDMAs), located at the N-terminal site to activate them [109,121,138]. However, in specific cases, the interaction between Argonaute proteins and some Tudor proteins is independent from the sDMAs, as demonstrated for the interaction between Aubergine and Kumo [120] or for Ago3 and Krimper [89]. In the case of dFmr1 and Aubergine, it seems more likely that the interaction occurs independently from the sDMAs residues of Aubergine because it has been demonstrated that the biochemical interaction of the two proteins is not limited to the N-terminal domain of Aubergine [105].

### 3.2. dFmr1’s Role in the Ovary

Recently, dFmr1 has emerged as a member of piRNA-mediated pathways in ovaries [105,139]. The first indication of the involvement of dFmr1 in the piRNA pathway of the ovary was the activation of transposons caused by the loss of dFmr1. The level of transcription of specific transposons increases significantly in the *dFmr1* mutant ovaries accompanied, as expected, by the reduction of fertility. Moreover, it is interesting to note that the expression patterns of dFmr1 and the piRNA-related protein Aubergine co-localize in specific territories of the ovary [105].

The *Drosophila* ovary is composed of 12 to 16 ovarioles. In each one, the most apical structure is the germarium, where stem cells are located. The developing egg chamber passes through the germarium and proceeds through its development, forming a linear chain. dFmr1 and Aubergine co-localize in the germarium, where they accumulate at the position of the stem cells. The two proteins also co-localize in the nuage of the nurse cells and, in the later stages, they overlap in the cytoplasm of the oocyte [105]. The relationship between dFmr1 and the piRNA pathway is also confirmed by the interaction between dFmr1 and Piwi, which act together in the heterochromatic gene silencing in somatic cells and the transposon silencing in germ cells [140]. In particular, it has been demonstrated that dFmr1 is essential for the correct localization of HP1, in the heterochromatin of follicle cells [139]. It is known that HP1 is a highly conserved protein identified as a crucial component of the heterochromatin with a role in the Position Effect Variegation (PEV), now also recognized as playing roles in many other processes [141].

In the ovary, dFmr1 is also involved in the microRNA-mediated pathway. The tight link between dFMR1 and the miRNA pathway has emerged from the interaction between the Fragile-X protein and the *bantam* miRNA to control germline stem cells in the ovary of *Drosophila* [142]. In human cell lines, FMRP interacts with components of the miRNA pathway, such as Dicer1 and Ago1 [143]. In *Drosophila*, Ago1 is also required for the biological function of dFmr1 in neural development and synaptogenesis [9].

## 4. Genetic and Biochemical Interactors of dFmr1

In the last part of this review we will examine the multiple roles and functions of the dFmr1 protein, following the threads of its genetic and biochemical/physical interactors in the gonads and in the germ granules representing a bridge from oocyte to embryos in the fly and other animals (mice and humans).

dFmr1 has recently been associated with the piRNA pathway in the gonads of both sexes [105,139] and the interaction with some of the Argonaute proteins supports these findings. dFmr1 interacts with Aubergine, allowing the correct silencing of the transposons and repetitive sequences [105], and with Piwi [139], cooperating in the piRNA-mediated translational silencing of the transposons. These two proteins belong to the Piwi-subfamily of the Argonaute proteins, and whereas Aubergine is cytoplasmic as well as the most common localization of dFmr1, Piwi is predominantly nuclear, confirming a nuclear function and localization of dFmr1. Ago1, belonging to the Ago-subfamily of the Argonaute proteins, is a biochemical and genetic interactor of dFmr1 in the piRNA pathway [105], as well as in the miRNA pathway in the ovary [9,142,143,144,145,146,147].

The DEAD box RNA helicase Vasa is also considered a key component of the piRNA pathway; it is located at the nuage of ovaries and testes [35,51,83,134,148,149,150,151,152] and has been considered a ‘‘biochemical platform’’ to put together the key components of the piRNA amplification machinery (the Amplifier complex) in nuage [86]. dFmr1 has been demonstrated to co-localize with Vasa in the testes and ovaries [105]. The function of the dFmr1 gene is also required for pole cells formation, located at the posterior pole of the early embryo, in which the Vasa protein is one of the main components of the polar granules [134,153,154]. These findings support the hypothesis that dFmr1 and Vasa may exert their role in the same pathway from the germ cells in the ovary to the pole cells in the embryo in the silencing of the transposable elements maintaining genome stability in these tissues. 

Another component of the germ granules at the pole plasm, where the pole cells will form, is the RNA binding protein Cup [155,156,157]. The gene coding for Cup has been found in a genetic screening for interactors of dFmr1 [158]. It codes for an RNA binding protein promoting the accumulation of the germ plasm components like Oskar, Vasa and Staufen at the posterior pole of the oocyte and it is required for the correct polar granules deposition [157,159]. During the development of the early stages of oogenesis, Cup co-localizes and interacts with Hsp83 [157]. Hsp83 has a well-demonstrated role in the silencing of transposons and repetitive sequences related to the genome stability [95,101,160,161,162].

The P-bodies component, Me31B DEAD box helicase, represents a very intriguing link between Cup, Hsp83 and dFmr1 [163]. Me31B co-localizes with dFmr1 and Cup in these cytoplasmic structures implicated in RNA metabolism like storage, translational repression, and RNA degradation. Me31B also interacts with Aubergine and Vasa, and is required to recruit dFmr1 to the neuronal granules [164,165,166,167], which may be considered a neuronal counterpart of the polar granules.

An emerging function which has been demonstrated for the mammalian form of Fmr1 protein is related to its capacity to recognize the G-quadruplets, a specific structure in RNAs [168]. It is interesting to note that the G-quadruplets have been shown to be present in the precursors of the piRNAs [47]. The connection between the *Drosophila* dFmr1 and the G-quadruplets can be supported by the finding that one of the targets of dFmr1 in the nervous system is the protein Futsch, the homolog of the mammalian MAP1B, exhibiting a putative G-quadruplets in its 5′ end [19,144,153]. This specific aspect has not yet been completely elucidated in *Drosophila*.

### Nuclear Interactors in the Small RNA Pathway (in the Gonads)

In addition to the cytoplasmic function of dFmr1, a nuclear function has been suggested due not only to the NLS and NES domains in the protein (Figure 1) [1,24,169], but also to some of its nuclear interactors. One of the known nuclear interactors in mammals is NUFIP (Nuclear Fmr1 Interacting Protein), a nucleo-cytoplasmic shuttling protein related to the ribonucleoprotein (RNP) complex formation [170,171,172]. Recently dFmr1 and Nufip have been identified in a Zfrp8 complex in the *Drosophila* ovary [137]. Zfrp8 participates in the piRNA pathway, influencing the correct localization of Maelstrom in the nuage [173], and is required for maintaining follicle and germline stem cells (GSCs) [174]; it has been proposed that Zfrp8 is required in order to localize FMRP properly. Interestingly Hsp83, Hop (Hsp70/Hsp90 Organizing Protein Homolog), and Piwi have been identified in the same complex with a hypothesized role in piRNA-mediated canalization [137,175,176].

The emerging role of dFmr1 in the nucleus is strongly supported by its connection with HP1. In the *Drosophila* ovary, the loss of dFmr1 causes an incorrect localization of HP1 in the heterochromatin, suggesting a role for dFmr1 in chromatin state maintenance [139].

## 5. Conclusions

dFmr1 is an RNA binding protein with multiple domains and multiple demonstrated functions. Some of these functions may be related to the different interactors in different tissues or in different subcellular populations, or both. In this review, following its interactors in the gonads, cells, and subcellular compartments, we have analyzed dFmr1’s roles in small RNA pathways: in regulating the localization of some nuage components, in shuttling between nucleus and cytoplasm, in chromatin remodeling, in binding the G-quadruplets and, finally, in potentially regulating mRNA translation in the ovary as well as at the synapses. All these studies indicate a conservation of the pathways involved in the regulation of transposons from the animal models to humans, and furthermore emphasize the importance of the *Drosophila* model for the discovery of new components of the piRNA pathway as well as for molecular studies of the piRNA process to be applied in mammals. 

It will be interesting, in the future, to further investigate the different roles of FMRP arising from its multiple interactors in different types of cells and tissues, in order to gain insights into its apparently different molecular roles that have a significant impact on human Fragile-X disease.

## Figures and Tables

**Table 1:** piRNA-related genes and the *crystal-Stellate* regulation. The best-known piRNA genes, with their domains and localizations reported. Abbreviations: N = nuclear; C = cytoplasmic; ND = not determined; the asterisks (*) indicate that the protein is located at the piNG bodies; “+” indicates the presence of the protein and/or of the crystals; “++” indicates a high presence of the protein and/or of the crystals; “++++” indicates a very high presence of the protein and/or of the crystals, “++”, where indicated the information are on the RNA coding for crystals; “?” indicates that the information are not available.

Mutants	Protein Domains	Protein Localization	Testes	Ovaries	Protein Function	Transposons	Stellate-Made Crystals	References
	**Argonaute Proteins**							
*aubergine*	PAZ, MID, PIWI	germline (C-nuage); germ granules	+ *	+	ping-pong pathway, primary pathway	germinal	+	[35,38,78,83,88,108]
*ago3*	PAZ, MID, PIWI	germline (C-nuage); germ granules	+ *	+	ping-pong pathway	germinal	+	[35,70,71]
*piwi*	PAZ, MID, PIWI	soma + germline (nuclear)	+	+	primary pathway	germinal + somatic	-	[83,84,109]
*ago1*	PAZ, PIWI	soma, germline (C-nuage)	+ *	+	piRNA pathway, miRNA pathway	somatic	+	[105,111]
	**Helicases**							
*armitage*	RNA helicase (SDE3)	soma(C-Yb bodies) + germline (C)	+	+	primary pathway	germinal + somatic	+	[70,71,112,113,114]
*vasa*	DEAD RNA helicase	germline (Amplifier Complex-C-nuage); germ granules	+ *	+	primary + ping-pong pathway	germinal + somatic	++ (RNA)	[84,86]
*UAP56*	DEAD RNA helicase	germline (N) + N/C boundary	ND	+	primary + ping-pong pathway	germinal + somatic	ND	[73,75]
	**Tudor Proteins**							
*Yb*	DEAD RNA helicase + Tudor domain	soma (C-Yb bodies)	ND	+	primary pathway (single strand clusters (flamenco))	somatic	-	[32,70,71,113,114]
*spindle-E*	DEAD RNA helicase + Tudor domain	germline (C-nuage)	+ *	+	primary pathway	germinal	+	[61,84,104]
*krimper*	Tudor domain	germline (C-nuage) + soma (krimper body)	+	+		germinal	++++	[61,115]
*tudor*	Tudor domain	germline (C-nuage) soma; germ granules	+ *	+		germinal	-	[116,117,118]
*tapas*	Tudor domain + Lotus	germline (C-nuage)	+	+	ping-pong?, primary?	germinal	++++ (RNA)	[113,119]
*tejas*	Tudor domain + Lotus domain	germline (C-nuage)	+	+	ping-pong?, primary?	germinal	++++	[84]
*zucchini*	Tudor domain + nuclease	soma(C) + germline (C-nuage) mitocondrial	+	+	primary pathway	germinal + somatic	+	[65,66,69,71,81]
*squash*	Tudor domain + nuclease	germline (C-nuage); ND in the soma	+ *	+		germinal + somatic	+	[69,81]
*vreteno*	Tudor domain + RRM domai	soma (C) + germline (C-nuage)	+	+	primary pathway	germinal (seen only in ovaries) + somatic	ND	[113,114]
*qin/kumo*	Tudor domain + RING domain	soma (N) + germline (Amplifier Complex-C-nuage)	+	+	primary pathway (dual strand clusters)	germline + somatic	+	[75,85,120]
*dFmr1*	Tudor domain, KH, RGG, NLS, NES	soma + germline (N + C)	+	+		germinal + somatic	+	[105]
*papi*	Tudor domain + KH domain	soma (N) + germline (C-nuage)	ND	+	primary + ping-pong pathway	germinal + somatic	ND	[121]
*eggless (dSETDB1)*	Tudor domain + SET domain	germline (N) + soma (N)	ND	+	H3K9 methyl transferase (TE regulation)	germinal + somatic	ND	[122]
	**Non Tudor Proteins**							
*rhino*	chromo/shadow domain	germline + soma (N)	ND	+	primary + ping-pong pathway (dual strand clusters)	germinal	-	[57,76,82]
*cutoff*	DXO/Dom3Z family	germline (C-nuage) + N	ND	+	primary + ping-pong pathway (dual strand clusters)	germinal	-	[57,76]
*maelstrom*	Maelstrom domain (DNA/RNA binding); HMG protein	germline + soma: shuttle nucleus-cytoplasm (nuage)	+	+	primary + ping-pong pathway	germinal + somatic	ND	[123]
*capsuleen*	PRMT	germline (C-nuage)	+	+	arginine methyltransferase	germline	+	[124,125]
*shutdown*	FKBP6 family (co-chaperone)	soma (YB-bodies) + germline (N + C)	+	+	primary pathway + ping-pong	germinal + somatic	+	[115,127]
*hsp83*	heat shock protein	soma + germline (N + C)	+	+	primary pathway + ping-pong	germinal + somatic	+	[70,78,99,101]
